# The Twelve Tissue Remedies of Schüssler

**Published:** 1893-05

**Authors:** 


The Twelve Tissue Remedies of Schussler, Comprising the Theory, Therapeutical Application, Materia Medica, and a Complete Repertory of these Remedies, Homœopathically and Bio-chemically Considered. By Wm. Bœricke, M. D., and Willis A. Dewey, M. D. Third edition, rewritten and enlarged. Philadelphia : Bœricke & Tafel, 1893. Price, $2.50 net.

This book has been regularly reviewed by The Homœopathic Physician as each edition, by whomsoever edited, has appeared. It has never been commended by this journal. The objections to it have always been plainly stated, and it may not be amiss to repeat two of them here. In the first place, it was attempted by the author, Schüssler, to reduce the resources of the materia medica to twelve remedies. Thus a large and valuable collection of remedies was discarded and the labor of prescribing abridged. It was a case of prescribing made easy.

To justify such a limited range of therapeutic agents, the facts of chemistry and physiology, and of the homœopathic provings were freely used to weave a web of pathology and rationalism, and the fabric so woven is called in the latest edition of the book "Bio-chemistry." The late Dr. Lippe used to say that this fabric had been made up into "the pathological livery."

In the second place, it was attempted to substitute for the labor of searching the materia medica for the proper medicine, the easy process of "reasoning" out the remedy. This was, of course, a nullification of Homœopathy.

In the volume now under notice, the remedies as given by Schüssler are so expanded and improved by the addition of genuine "guiding symptoms" taken from the homœopathic *Materia Medica*, that Schüssler's speculations are very much obscured, if not lost sight of altogether. The present editors, in their preface, significantly remark: "We believe that the only hope for the future development of these magnificent remedies lies in their study, mainly according to the method of Homœopathy; that they should all be as carefully proved as Natrum-mur. and Silicea already are, and that the results of such provings alone will furnish the most accurate indications for their therapeutical uses. Only by careful provings will the permanency of these remedies be secured, and they themselves be preserved from the possible fate of so many newly introduced remedies." In this avowal of the editors is a virtual abandonment of "Schüsslerism," and in their levying upon the genuine materia medica for symptomatology they actually render Schüssler's theories inoperative in practice. They thus show their good sense and their regard for Homœopathy and at the same time render the book far more valuable. While we are not yet satisfied with the volume as it stands, still the enlightened work which they have put upon it compels us to withdraw much of the objection with which we have heretofore greeted it in these pages. Schüsslerism in its purity leads the mind infallibly back to the rational therapeutics of the old school, from which it was rescued by Homœopathy. Therefore Schüsslerism should be relegated to oblivion. The editing of the present volume shows that it is "getting there."

